# Supplementary material for: Cloud BioLinux: pre-configured and on-demand bioinformatics computing for the genomics community
Source: BMC Bioinformatics. 2012 Mar 19;13:42. doi: 10.1186/1471-2105-13-42 (PMC3372431; doi:10.1186/1471-2105-13-42)
Supplement: Additional file 1 — Supplementary 1 Cloud BioLinux software documentation in the form of a mini, self-contained website. Users need to download and uncompress the .zip file, and open through a web browser the "index.html" file available on the main directory. (ZIP 1823 kb). [file 1471-2105-13-42-S1.ZIP › Cloud-BioLinux-Package-Documentation/docs/seqboot.html]

Bio-Linux Software Documentation Pages

Back to search form

## seqboot

|  |  |
| --- | --- |
| Name | seqboot |
| Description | **seqboot** is part of the PHYLIP package  Copyright 1991-2004 by the University of Washington. Written by Joseph Felsenstein. Permission is granted to copy this document provided that no fee is charged for it and that this copyright notice is not removed.  **seqboot** is a general bootstrapping and data set translation tool. It is intended to allow you to generate multiple data sets that are resampled versions of the input data set. Since almost all programs in the package can analyze these multiple data sets, this allows almost anything in this package to be bootstrapped, jackknifed, or permuted.  **seqboot** can handle molecular sequences, binary characters, restriction sites, or gene frequencies. It can also convert data sets between Sequential and Interleaved format, and into the NEXUS format or into a new XML sequence alignment format.  **References:**  Felsenstein, J. 1993. PHYLIP (Phylogeny Inference Package) version 3.5c. Distributed by the author. Department of Genetics, University of Washington, Seattle.    Felsenstein, J. 1989. PHYLIP -- Phylogeny Inference Package (Version 3.2). Cladistics 5: 164-166. |
| Homepage | http://evolution.genetics.washington.edu/phylip.html |
| Remote Documentation | http://evolution.genetics.washington.edu/phylip/doc/seqboot.html |
